# A Complicated Case of Innominate Artery Avulsion and Subsequent Aorto-subclavian Graft Occlusion

**DOI:** 10.7759/cureus.5056

**Published:** 2019-07-01

**Authors:** Mansoor Ahmad, Muhammad Asghar, Chirag Divecha, Timothy W Swain

**Affiliations:** 1 Internal Medicine, University of Illinois College of Medicine at Peoria, Peoria, USA; 2 Cardiology, University of Illinois College of Medicine at Peoria, Peoria, USA; 3 Cardiac Surgery, University of Illinois College of Medicine at Peoria, Peoria, USA

**Keywords:** innominate artery avulsion, aorto-axillary graft

## Abstract

A 41-year-old female presented with complaints of right arm claudication, weakness, and pain associated with serous drainage from a previous incision site to the right anterior chest. At age 16, this patient was involved in a motor vehicle accident, which resulted in a right innominate artery and brachiocephalic vein avulsion. The two vessels were immediately ligated and oversewn. The perfusion to her right arm was supplied by cerebral collateral circulation down the right vertebral to the right subclavian artery.

## Introduction

It is quite uncommon to encounter difficult cases of innominate artery repair via an aorto-subclavian graft. We are reporting a complicated case of innominate artery avulsion (secondary to motor vehicle accident) and repair via an aortic to subclavian arterial Gore-Tex (W. L. Gore & Associates, Inc., Arizona, US) bypass graft with subsequent occlusion and re-grafting, after 25 years of initial grafting.

## Case presentation

A 41-year-old female presented with complaints of right arm claudication, weakness, and pain associated with serous drainage from a previous incision site to the right anterior chest. At age 16, this patient was involved in a motor vehicle accident, which resulted in a right innominate artery and brachiocephalic vein avulsion. The two vessels were immediately ligated and oversewn. The perfusion to her right arm was supplied by cerebral collateral circulation down the right vertebral to the right subclavian artery.

The patient was stable until the age of 35 years when symptoms of ischemia set in and she developed right arm pain. This led to her readjusting her right arm frequently and an inability to sleep secondary to ischemic pain. It required surgical intervention done at the University of Chicago with the placement of a right aortic to axillary bypass graft.

**Figure 1:**
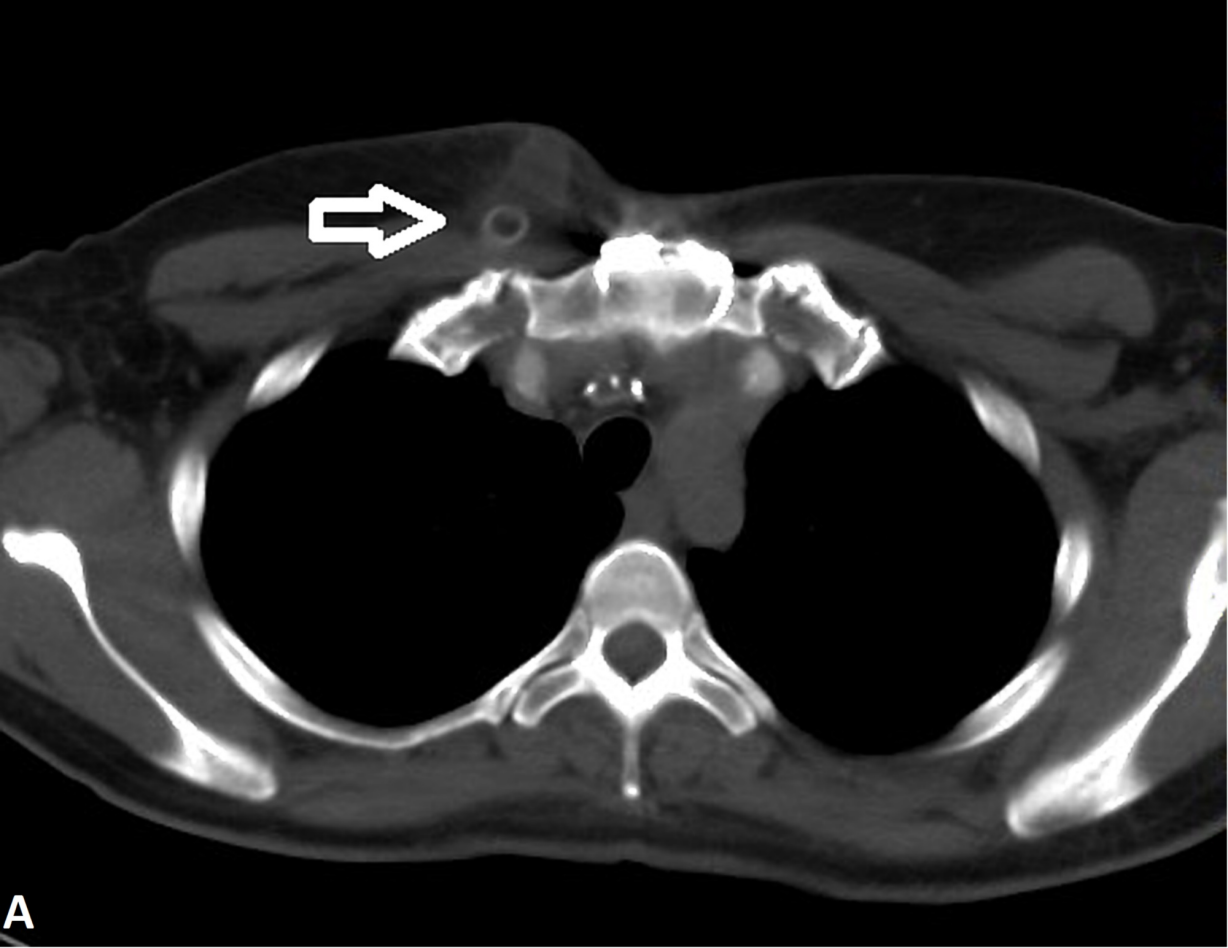
CT angiogram of the chest showing an area of fluid collection in the right paramedian subcutaneous anterior chest wall adjacent to the anterior aspect of the occluded aorto-axillary graft (arrow). This represented seroma or lymphocele. There is adjacent fat stranding, which may represent the scarring of cellulitis.

Six years passed before the patient began developing her recent symptoms of right arm claudication and weakness. Upon the patient’s recent presentation, a computed tomography (CT) angiogram of this graft showed occlusion of the aortic-axillary bypass graft due to thrombosis. Interventional radiology decided to thrombolyse the clot and re-canalize the graft. However, the patient developed an infection of the graft with serous discharge, bacteremia, and persistent symptoms of right upper extremity arterial occlusion (Figure 1).

The persistence of these symptoms and the infection of the graft led to the decision to perform a two-staged procedure: 1) removal of the infected/occluded aorto-subclavian bypass graft with the exception of the aortic stump; this was performed without sternotomy. The patient was treated with antibiotics and once the infection was cleared, the next stage involved redo sternotomy with 2) removal of the old aorto-axillary graft stump and creation of a new aorta to right subclavian artery stump with a direct path using an open harvest of a piece of the left greater saphenous vein.

Procedure

The previous incision over the right anterior chest was reopened. The sternum was not opened at this time. The graft was controlled and the subclavian artery was exposed. A large number of adhesions was noted, requiring extreme care to liberate and identify the right subclavian artery and vein. The patient was heparinized using 8000 units. The left greater saphenous vein was harvested open. Once the subclavian artery was isolated, all graft material was removed up to the incorporated graft material just medial to the aorta. A remnant of the graft was left attached to the lateral position of the aorta, as it appeared to be incorporated into the surrounding tissue. The laterally surrounding tissue was noted to have induration but no gross pus present. However, once the lateral graft was incised, pus spilled out. All material was sent for culture. Proximal and distal control of the subclavian artery was obtained and the defect of the arterial wall was closed using a saphenous venous patch and 6-0 Prolene sutures. The distal flow was noted following repair and clamp removal. Hemostasis was obtained. Due to active infection, it was deemed unsafe to remove the stump and re-implant another graft. The incision was left open and a wound vacuum was placed medially. The incision over the axillary artery was loosely closed and a drain was placed.

Post-operative care and follow-up procedure

The drain was removed at three days post-procedure. Home wound vacuum and intravenous antibiotics were initially started with vancomycin for two weeks followed by Bactrim DS twice daily for two weeks. The infected graft and pus were tested for aerobic, anaerobic, and fungal cultures. The patient did not show signs of infection after the antibiotics (Table 1).

**Table 1:** Post-operative laboratory work WBC: white blood cells, HB: hemoglobin, HCT: hematocrit, PLT: platelets

	11/15/18	11/20/19	01/18/19	01/20/19	01/28/19
WBC	5.52	5.7	9.81	6.00	6.44
HB	9.5	11.3	10.6	10.4	10.7
HCT	29.9	35.8	33.9	33.4	34.9
PLT	117	157	140	132	247

On follow-up after the first staged procedure, the patient denied fever, chills, weight loss, or any other constitutional symptoms. There was excellent granulation tissue, and the incision was approximately 50% closed with no surrounding erythema, odor, drainage, or fluctuance. The incision subsequently healed and the wound vacuum was removed.

Two months after the initial procedure to remove the graft, the patient underwent redo sternotomy to remove the remainder of the old graft. During this same procedure, a right subclavian to aorta graft was placed using an 8 mm Gore-Tex graft (W. L. Gore & Associates, Inc., Arizona, US). This procedure resulted in total relief of the symptoms. The patient continues to do well.

## Discussion

The first successful repair of an innominate artery injury after blunt trauma was reported by Binet in 1962 [1]. The innominate artery is fixed at the origin from the aortic arch with the distal portion of the artery free and mobile, which results in injury to the artery, mostly at the origin [2-3]. Motor vehicle accidents result in deceleration injuries and compression against steering wheels, which also results in such a pattern of injuries [2]. The innominate artery seems most vulnerable of all aortic branches, usually from avulsion injuries or by direct compression between the sternum and vertebral bodies [4].

Various surgical options are available to deal with such an innominate artery blunt trauma. If the laceration is close to the origin, bypass grafting can be done from the ascending aorta to the distal part of the axillary artery. If the injury is to the mid-region, interposition grafting can be done. If there is no extensive tissue loss, a primary repair can be done [5]. A cardiopulmonary bypass with hypothermia and selective cerebral perfusion has been also reported to be useful in cases with extensive injury or uncontrollable hemorrhage. In patients with stable hemodynamics, surgical treatment without a cardiopulmonary bypass has also been used [6-7]. Freeman and Leeds introduced the concept of the extra-anatomic bypass in 1952 [8]. They used a superficial femoral artery to carry blood from one femoral artery to another. This procedure is now an accepted method of revascularization.

In our patient, aorto-subclavian bypass grafting was initially performed for innominate artery avulsion after a motor vehicle accident occurring 26 years prior in 1992. The initial graft was patent for 20 years prior to occlusion (2011). The patient developed occlusive symptoms rather early, for which interventional radiology attempted clot thrombolysis, but the procedure was unsuccessful. This resulted in persistent occlusion symptoms and was later complicated with a chronic serous infection of the graft. Removal of the graft, repair of the subclavian artery, and re-grafting was performed, which led to the resolution of symptoms.

Graft stenosis can be asymptomatic or have symptoms similar to those in patients with subclavian or innominate artery stenosis due to atherosclerosis. These patients may present with symptoms of vertebrobasilar ischemia, including episodes of dizziness, diplopia, ataxia, vertigo, limb claudication, paresthesia, and steal syndrome. Physical examination may reveal diminished pulse and decreased blood pressure (>20 mmHg reduction as compared to the opposing extremity) in the affected limb.

In this case, the graft was occluded and infected, which required the removal of the infected graft and subsequent re-grafting. Re-grafting posed more challenges due to multiple adhesions from previous procedures. Exposing the subclavian artery is associated with risks of possible complications, such as injuries to the phrenic nerve, the recurrent laryngeal nerve, the vagus nerve, the cervical sympathetic chain, and the thoracic duct, which are located in the vicinity of the subclavian artery.

## Conclusions

An aorto-subclavian bypass graft is an uncommon, but effective, way of repairing an innominate artery injury subsequent to blunt trauma resulting in avulsion. Long-term complications may include graft occlusion requiring re-grafting. All measures should be taken to avoid infection postoperatively.
